# Evaluating the disease and treatment information provided to patients with chronic obstructive pulmonary disease at the time of discharge according to GOLD discharge guidelines

**DOI:** 10.31744/einstein_journal/2020AO4706

**Published:** 2019-12-03

**Authors:** Letícia de Araújo Morais, Samylla Ysmarrane Ismail Eisha de Sousa Cavalcante, Marcus Barreto Conde, Marcelo Fouad Rabahi

**Affiliations:** 1 Universidade Federal de Goiás , Goiânia , GO , Brazil .; 2 Universidade Estadual de Goiás , Anápolis , GO , Brazil .; 3 Universidade Federal do Rio de Janeiro , Rio de Janeiro , RJ , Brazil .

**Keywords:** Pulmonary disease, chronic obstructive, Hospitalization, Patient discharge

## Abstract

**Objective:**

To evaluate the disease and treatment information provided to patients with chronic obstructive pulmonary disease at hospital discharge.

**Methods:**

This was a cross-sectional study including hospitalized patients with chronic obstructive pulmonary disease at three tertiary hospitals. The study was based on seven items of the Global Initiative for Obstructive Lung Disease (GOLD) discharge guidelines. Two hospitals in this study had a Medical Residency Program in Pulmonology, and one did not have the program.

**Results:**

Fifty-four patients were evaluated. Large amounts of information were provided concerning effective pharmacological maintenance (item 1), blood gas evaluation/measurement of oxygen saturation (item 2), assessment of inhalation technique (item 4), and maintenance therapy (item 5). Less information was provided regarding comorbidity management planning (item 3), the completion of antibiotic/corticosteroid therapy (item 6) and follow-up with the attending physician or specialist (item 7) had less information. We observed significant differences between hospitals for items 1, 4 and 7, and better performance in hospitals with medical residency in pulmonology.

**Conclusion:**

Hospitalized patients with chronic obstructive pulmonary disease received little to no information about the seven items addressed by GOLD discharge guidelines. This finding suggests that these guidelines should be used more often by clinicians in hospital with or without medical residency in pulmonology. The lack of specialized care resulted in insufficient amount of information for patients with chronic obstructive pulmonary disease at discharge.

## INTRODUCTION

Chronic obstructive pulmonary disease (COPD) is a heterogeneous and multifactorial clinical condition with estimated global prevalence of 11.7%. In 2012, COPD had caused more than 3 million deaths, which accounted for 6% of all deaths globally in that year. ^1^ Because COPD is a disease with many national and international guidelines, patients with this disease are expected to receive standard treatment. Chronic obstructive pulmonary disease is a chronic condition and requires frequent contact with physician, and patients need to be aware of relevant information regarding the disease and its treatment.

Exacerbations are the main cause of COPD-related hospitalization, and different variables are associated with a significant high number of early readmissions of these patients. ^2 - 5^ In 2014, the Global Initiative for Chronic Obstructive Lung Disease (GOLD) proposed guidelines that aimed to guarantee that COPD patients were evaluated, discharged, and followed-up appropriately after hospitalization due exacerbation. ^6^ These guidelines involve a checklist with seven items regarding home pharmacotherapy regimen, inhaler technique assessment, maintenance regimen, appropriate use of steroid therapy and antibiotics (when prescribed), long-term oxygen therapy requirement, a follow-up visit for 4 to 6 weeks, and a management plan for comorbidities and associated follow-ups, which should be discussed along with the patient. ^6^ The use of these guidelines among clinicians has been assumed to be associated with lower risk of clinical exacerbation and hospital readmissions. However, the use of the GOLD discharge guidelines is not routine in Brazilian hospitals.

## OBJECTIVE

To evaluate whether information about the disease and treatment, based on the Global Initiative for Chronic Obstructive Lung Disease discharge guidelines, was provided to patients with chronic obstructive pulmonary disease by their clinicians at discharge, and to compare the knowledge of these patients about this condition and its treatment.

## METHODS

### Setting

This was a cross-sectional study conducted in two hospitals with Medical Residency Program on Pulmonology ( *Hospital das Clínicas of Universidade Federal de Goiás,* CAAE: 43653515.1.0000.5078, under number 1.049.091 − and Hospital Estadual Geral de Goiânia Dr. Alberto Rassi, CAAE: 43653515.1.3001.0035, under number 1.109.145) and one hospital without a this program on pulmonology (Santa Casa de Misericórdia de Goiânia, CAAE: 43653515.1.3002.5081, parecer 1.123.580).

### Study period

#### Subject selection, data collection, and definitions

Between July 6, 2015 and November 4, 2015, we invited to participate patients aged 40 years or older who were admitted with a diagnosis of COPD as indicated by their physicians and who did not receive respiratory support nor exhibited cognitive or neurological impairment (as evaluated by the interviewer) at hospital discharge. After signing the Consent Form, participants enrolled in the study were interviewed by one researcher/interviewer trained for this purpose. The interviewer completed a data collection document in Portuguese created specifically for this study (the English version is presented in table 1 ). This document was based on the English version of the GOLD discharge guidelines, which was translated from English into Portuguese with the consent of its creators according to methods previously described. ^7 , 8^ The data collection allowed the interviewer to evaluate the amount of information about the disease and treatment provided to patients with COPD at discharge based on seven questions of seven items of the GOLD discharge guidelines. The data collected were tested, evaluated, and modified in a pilot including ten patients. The results were not included in this study. We did not inform clinicians about the purpose of this research.

**Table 1:** Individual data collection used to evaluate information about the disease and treatment provided to patients with chronic obstructive pulmonary disease at discharge based on Global Initiative for Chronic Obstructive Lung Disease discharge guidelines

Item	Procedures	Interpretation of the procedure	Answer (based on the interpretation of the procedure, choose “yes” or “no”)
1.Were the usual medications for COPD prescribed?	Evaluate the prescription given to the patient	If the prescription includes the use of at least one of the following inhaled medications: a short-acting beta-2 agonist; a long-acting beta-2 agonist alone or in combination with a corticosteroid; or a long-acting anticholinergic agent alone or in combination with previous medications, then the answer must be “yes”. If the patient does not indicate the use of the above medications, then the answer must be “no”	Yes No
2.Were arterial blood gases and SpO _2_ measurement performed?	Refer to the patient’s medical record to determine whether arterial blood gases and SpO _2_ measurement were performed	If an evaluation of one or both items was performed, then the answer will be “yes”. If no information is available regarding either measurement, then the answer is “no”	Yes No
3.Was a therapeutic proposal provided regarding a follow-up for comorbidities?	Refer to the patient’s medical record or communicate directly with the attending physician regarding whether a therapeutic proposal was provided with a follow-up for comorbidities	If a description is present in the medical record or a referral for a follow-up for comorbidities was provided, then the answer is “yes”. If no recommendation for an evaluation of comorbidities is present, then the answer is “no”	Yes No
4.Has the inhalation technique been assessed by the support team?	Ask the patient to describe the inhalation technique as defined by the health team	If the patient indicates that the inhalation technique was assessed by the support team, then the answer is “yes”. If the patient indicates that he was not approached regarding inhaled medications or that he is not using inhaled medications, then the answer is “no”	Yes No
5.Has the patient been informed about the proposed medical treatment?	Ask the patient whether he was informed of the importance of maintaining the proposed medical treatment	If the patient received information about the need for continued medication use, then the answer is “yes”. If the patient did not receive information about the need for continued medication use, then the answer is “no”	Yes No
6.Was information provided regarding the treatment period for corticosteroid and antibiotic medications?	Ask the patient or the treating physician whether the patient was informed about the duration of corticosteroid or antibiotic medication use, if prescribed	If instructions were written on the discharge prescription or the patient was clearly told that correct use of antibiotic and corticosteroid medications was important, then the answer is “yes”. If the aforementioned information was not relayed, then the answer is “no”	Yes No
7.Was a follow-up scheduled with the attending physician or a pulmonologist?	Ask the patient whether he has a follow-up appointment scheduled with the attending physician or a pulmonologist after discharge	If a definite follow-up visit with the attending physician or a pulmonologist is scheduled, then the answer is “yes”. If no follow-up appointment is scheduled, then the answer is “no”	Yes No

COPD: chronic obstructive pulmonary disease; SpO _2_ : oxygen saturation.

Source: Based on Morais LA, Conde MB, Rabahi MF. Avaliação padronizada de pacientes com DPOC no momento da alta hospitalar. Rev Educ Saude. 2016;4(2):88-94; Chart 3: Standard form for verification of items proposed by GOLD in COPD patients at the time of hospital discharge.

Patients who did not complete the study procedures were excluded. After interviewing of all participants, all clinicians were interviewed about the use of GOLD discharge guidelines.

Data were analyzed using the (SPSS), version 23. A significance level of 5% (p<0.05) was adopted in the analyses. The Shapiro-Wilk test was used to assess the distribution (normality) of numerical variables. Sociodemographic data and responses in the document were characterized by absolute and relative frequencies. χ ^2^ test or Fisher’s exact test was used to evaluate qualitative variables as appropriate. The means for quantitative variables were compared using *t* -tests. A reliability test was conducted for data collection using the kappa coefficient.

## RESULTS

Sixty-three patients were eligible for the study. Of these, nine refused to sign the informed consent form. The final sample was composed by 54 subjects (22 from the hospitals with a Medical Residency Program on Pulmonology and 32 from the hospital without the program). The sociodemographic characteristics of participants are shown in table 2 . Among the 12 clinicians interviewed about the use of the GOLD guidelines at discharged, all denied its use.

**Table 2:** Sociodemographic characteristics of the study participants

Characteristic	Total n=54	RPP n=22	No RPP n=32	p value
Age, years	66.6±11.4	63.5±12.0	68.7±8.6	0.09
Male	28 (51.9)	14 (61.9)	11 (34.4)	0.06
Education				0.1
No schooling	8 (24.2)	6 (33.3)	2 (15.3)	
Fundamental	25 (75.8)	12 (66.7)	13 (86.7)	
Previous admission to the ICU	8 (14.8)	5 (19.0)	3 (9.4)	0.08
Readmission	14 (42.4)	8 (44.4)	6 (40.0)	0.8

Results expressed as mean±standard deviation, or n (%).

RPP: residency program in pulmonology; ICU: Intensive care unit.

As shown in table 3 , 75% (116/154) of the answers related with the seven questions were adequate among patients discharged from the hospitals with an Residency Program in Pulmonology, and 47% (106/224) were adequate among patients discharged from the hospital without a program. We observed significant differences between groups for items 1, 4 and 7.

**Table 3:** Disease and treatment information provided to the patients with chronic obstructive pulmonary disease at discharge

Item evaluated	RPP Yes n=22 n (%)	No RPP Yes n=32 n(%)	p value
1.Prescription for usual COPD medications	22 (100)	21 (66)	0.002*
2.Assessment of Arterial blood gases and SpO _2_	22 (100)	30 (94)	0.2
3.Management plan for comorbidities	6 (27)	12 (37)	0.4
4.Assessment of technique for inhaler use	20 (90)	18 (56)	0.001*
5.Information regarding maintenance therapy	19 (86)	29 (90)	0.6
6.Instructions regarding the duration of corticoid or antibiotic therapy	7(32)	15 (47)	0.2
7.Follow-up plan with a specialist	20 (90)	10 (32)	0.001*

* significant.

RPP: residency program in pulmonology; COPD: chronic obstructive pulmonary disease; SpO _2_ : oxygen saturation.

## DISCUSSION

Our study findings show that answers regarding the disease and treatment information provided to patients with COPD at discharge based on the GOLD discharge guidelines were, in general, more correct among those assisted at hospitals with a Residency Program in Pulmonology (75%) than those assisted at a hospital without the program (47%). The lowest levels of information provided by the hospitals with a Residency Program in Pulmonology, according to the patients and clinical pictures, were observed for item 3 (management plan for comorbidities) and item 6 (instructions regarding the duration of corticoid or antibiotic therapy). In hospitals without the program, the lowest levels of information were observed in items 3 (management plan for comorbidities), 4 (assessment of the technique for inhaler use), 6 (instructions regarding the duration of corticoid or antibiotic therapy), and 7 (follow-up plan with a specialist). These recommendations were raised by the GOLD discharge guidelines issued in 2014, and they were maintained and updated in the guideline 2019 version.

Physicians interviewed were medical residents who were responsible to attend patients in the ambulatory. The adherence to protocols for achieve a more direct and standardized patient care for all medical categories was strongly encouraged.

Chronic obstructive pulmonary disease is associated with comorbidities such as cor pulmonale and metabolic diseases that significantly influence prognosis. ^9^ Spielmanns et al., ^10^ showed that comorbidities may influence the risks of intensive care unit admission and mechanical ventilation. A total of 73% of patients from the Residency Program in Pulmonology hospitals and 63% from patients from the no residency Residency Program in Pulmonology hospital had no comorbidity management plans. The lack of an appropriate management plan for comorbidities may have a negative impact on treatment because patients hospitalized with COPD often have comorbidities that lead to worse outcomes than hospitalized patients without COPD. ^11^ A study had showed that readmissions may be avoidable if comorbidities are better evaluated during the hospital discharge transition. ^12^

Chronic obstructive pulmonary disease treatment depends on the use of multi-dose inhalers. ^13^ In our study, 44% of patients from no Residency Program in Pulmonology hospital and 10% of patients from the Residency Program in Pulmonology hospital were not familiar with the correct use of inhalers. According to a study conducted in Texas, previous use of dose inhalers reduced the hospital readmission rate by 9% after 30 days and 23% after 60 days, when COPD patients received correct instruction at discharge about the use of inhalers. ^13^

In total, 68% and 53% of the patients discharged from the Residency Program in Pulmonology and from the no Residency Program in Pulmonology hospitals received inadequate information regarding antibiotic and corticoid therapy, respectively. Evidence indicates that patients are often unable to recall their diagnoses or treatment plan at discharge. ^14^ This lack of information can lead to higher rates of hospital readmission and more problems during transition from inpatient to outpatient care. ^15^

In addition, 10% of the patients from the Residency Program in Pulmonology hospitals and 68% from the no Residency Program in Pulmonology hospital had no outpatient follow-up scheduled with a pulmonologist. The lack of follow-up is one of the variables associated with hospital readmission. ^16^ Parikh et al., demonstrated the value of providing an information bundle to COPD patients. ^17^ They found that 59.1% of patients returned to the outpatient clinic to visit a pulmonologist after receiving instructions to do so at hospital discharge.

A high proportion of patients (100% and 90%, respectively) from the Residency Program in Pulmonology and no Residency Program in Pulmonology hospitals were evaluated to determine the need for oxygen therapy by arterial blood gases evaluation, which is consistent with results reported by Carme et al. ^18^ A long-term oxygen therapy has shown effective results for reducing complications for patients with severe hypoxemic respiratory failure, although no differences were found in admission frequency or length of hospital stay before, during, or after oxygen therapy. ^19 , 20^

Our study has several limitations mainly related to the small sample size. The relatively short follow-up is also a limitation that should be considered in future studies. The results seem to be only applicable to patients with similar characteristics to those of our sample.

## CONCLUSION

The lack of information to patients with chronic obstructive pulmonary disease related to the seven items addressed by the Global Initiative for Chronic Obstructive Lung Disease guidelines provided at discharge suggests that these guidelines should be routinely used by clinicians in hospitals with and without Residency Program in Pulmonology. The true impact of this policy adoption should be evaluated using an interventional approach.
