# Supplementary material for: Evaluating the disease and treatment information provided to patients with chronic obstructive pulmonary disease at the time of discharge according to GOLD discharge guidelines
Source: Einstein (Sao Paulo). 2019 Dec 3;18:eAO4706. doi: 10.31744/einstein_journal/2020AO4706 (PMC6905159; doi:10.31744/einstein_journal/2020AO4706)
Supplement: Translation

# Translation: Avaliação da doença e informações sobre tratamento fornecidas a pacientes com doença pulmonar obstrutiva crônica no momento da alta, de acordo com diretrizes GOLD para alta hospitalar

**Authors:** Letícia de Araújo Morais, Samylla Ysmarrane Ismail Eisha de Sousa Cavalcante, Marcus Barreto Conde, Marcelo Fouad Rabahi

**Affiliations:** 1 Universidade Federal de Goiás, Goiânia, GO, Brasil .; 2 Universidade Estadual de Goiás, Anápolis, GO, Brasil .; 3 Universidade Federal do Rio de Janeiro, Rio de Janeiro, RJ, Brasil .

**Keywords:** Doença pulmonar obstrutiva crônica, Hospitalização, Alta do paciente

## Abstract

**Objetivo:**

Avaliar informações sobre a doença e o tratamento fornecidos a pacientes com doença pulmonar obstrutiva crônica no momento da alta hospitalar.

**Métodos:**

Estudo transversal incluindo pacientes internados com doença pulmonar obstrutiva crônica de três hospitais terciários. O estudo baseou-se em sete itens das diretrizes de alta hospitalar da *Global Initiative for Obstructive Lung Disease* (GOLD). Dois hospitais participantes deste estudo tinham um Programa de Residência Médica em Pneumologia, e o terceiro não tinha.

**Resultados:**

Foram avaliados 54 pacientes. Muitas informações foram fornecidas em relação à manutenção farmacológica efetiva (item 1), avaliação de gasometria/medida da saturação de oxigênio (item 2), avaliação da técnica de inalação (item 4) e terapia de manutenção (item 5). Foram fornecidas menos informações em relação ao planejamento do manejo de comorbidade (item 3), a realização de antibioticoterapia/corticoterapia (item 6) e seguimento com o médico assistente ou especialista (item 7). Observaram-se diferenças significativas entre os hospitais para os itens 1, 4 e 7, e melhor desempenho nos hospitais com Programa de Residência Médica em Pneumologia.

**Conclusão:**

Pacientes hospitalizados com doença pulmonar obstrutiva crônica receberam pouca ou nenhuma informação relacionada aos sete itens abordados pelas diretrizes da GOLD para alta. Esses achados sugerem que essas diretrizes deveriam ser utilizadas com maior frequência por clínicos em hospitais com ou sem Programa Residência Médica em Pneumologia. A falta de atendimento especializado resultou em informação insuficiente para pacientes com doença pulmonar obstrutiva crônica na alta hospitalar.

## INTRODUÇÃO

A doença pulmonar obstrutiva crônica (DPOC) é uma condição clínica heterogênea e multifatorial com prevalência global estimada de 11,7%. Em 2012, a DPOC foi responsável pela morte de mais de 3 milhões de indivíduos, representando 6% de todas as mortes em todo o mundo naquele ano.^1^Considerando que a DPOC é uma doença com diversas diretrizes nacionais e internacionais, espera-se que os pacientes afetados pela doença recebam tratamento padrão. Ainda, por se tratar de condição crônica e requerer contato constante com o médico, os pacientes precisam receber informações relevantes em relação à doença e ao seu tratamento.

As exacerbações são a principal causa de hospitalização pela DPOC, e diferentes variáveis são associadas com número significativamente alto de readmissões prematuras desses pacientes.^2 - 5^Em 2014, a *Global Initiative for Chronic Obstructive Lung Disease* (GOLD) propôs diretrizes com o objetivo de garantir que pacientes com DPOC fossem avaliados, recebessem alta e fossem acompanhados adequadamente após hospitalização causada por exacerbação.^6^Tais diretrizes envolvem *checklist* com sete itens relacionadas ao tratamento medicamentoso em domicílio, avaliação técnica de inalação, regime de manutenção, uso apropriado de tratamento com esteroides e antibióticos (quando prescritos), necessidade de oxigenoterapia de longo prazo, seguimento após visita de 4 a 6 semanas, e gerenciamento do plano para comorbidades e acompanhamentos associados, que devem ser discutidos com o paciente.^6^O uso destas diretrizes entre clínicos tem sido associado com baixo risco de exacerbações clínicas e readmissões hospitalares. Porém, a adoção das diretrizes GOLD de alta hospitalar não é rotina em hospitais brasileiros.

## OBJETIVO

Avaliar se a informação sobre a doença e o tratamento, baseada nas diretrizes *Global Initiative for Chronic Obstructive Lung Disease* de alta hospitalar, foi disponibilizada aos pacientes com doença pulmonar obstrutiva crônica pelos clínicos na alta hospitalar, e comparar o conhecimento destes pacientes sobre esta condição e seu tratamento.

## MÉTODOS

### Local do estudo

Estudo transversal conduzido em dois hospitais com Programa de Residência Médica em Pneumologia (Hospital das Clínicas da Universidade Federal de Goiás, CAAE: 43653515.1.0000.5078, parecer 1.049.091 − e Hospital Estadual Geral de Goiânia Dr. Alberto Rassi, CAAE: 43653515.1.3001.0035, parecer 1.109.145) e em um hospital que não possuía tal programa de residência (Santa Casa de Misericórdia de Goiânia, CAAE: 43653515.1.3002.5081, parecer 1.123.580).

### Período do estudo

#### Seleção dos participantes, coleta de dados e definições

Entre 6 de julho de 2015 e 4 de novembro de 2015, foram convidados para o estudo pacientes com idade ≥40 anos internados, com diagnóstico de DPOC e que não recebiam suporte respiratório e nem possuíam lesão neurológica ou cognitiva (como avaliada pelo entrevistador) na alta hospitalar. Após assinatura do Termo de Consentimento, os participantes do estudo foram entrevistados por pesquisador/entrevistador treinado para este objetivo. O entrevistador preencheu documento de coleta de dados em língua portuguesa criado especificamente para este estudo (a versão em inglês é apresentada na tabela 1 ). Esse documento foi traduzido da versão em inglês das diretrizes GOLD de alta hospitalar com autorização de seus criadores conforme os métodos anteriormente descrito.^7 , 8^O documento de coleta de dados permitiu que o entrevistador avaliasse a quantidade de informação da doença e do tratamento disponibilizada aos pacientes com DPOC na alta hospitalar por meio de sete questões baseadas em sete itens das diretrizes GOLD de alta hospitalar. A coleta de dados foi testada, avaliada e modificada em piloto incluindo dez pacientes. Os resultados não foram incluídos no estudo. O objetivo do estudo não foi informado aos médicos responsáveis pelo atendimento.

**Tabela 1:** Individual data collection used to evaluate information about the disease and treatment provided to patients with chronic obstructive pulmonary disease at discharge based on Global Initiative for Chronic Obstructive Lung Disease discharge guidelines

Item		Procedures	Interpretation of the procedure	Answer (based on the interpretation of the procedure, choose “yes” or “no”)
1.Were the usual medications for COPD prescribed?		Evaluate the prescription given to the patient	If the prescription includes the use of at least one of the following inhaled medications: a short-acting beta-2 agonist; a long-acting beta-2 agonist alone or in combination with a corticosteroid; or a long-acting anticholinergic agent alone or in combination with previous medications, then the answer must be “yes”. If the patient does not indicate the use of the above medications, then the answer must be “no”	Yes No
2.Were arterial blood gases and SpO_2_measurement performed?		Refer to the patient’s medical record to determine whether arterial blood gases and SpO_2_measurement were performed	If an evaluation of one or both items was performed, then the answer will be “yes”. If no information is available regarding either measurement, then the answer is “no”	Yes No
3.Was a therapeutic proposal provided regarding a follow-up for comorbidities?		Refer to the patient’s medical record or communicate directly with the attending physician regarding whether a therapeutic proposal was provided with a follow-up for comorbidities	If a description is present in the medical record or a referral for a follow-up for comorbidities was provided, then the answer is “yes”. If no recommendation for an evaluation of comorbidities is present, then the answer is “no”	Yes No
4.Has the inhalation technique been assessed by the support team?		Ask the patient to describe the inhalation technique as defined by the health team	If the patient indicates that the inhalation technique was assessed by the support team, then the answer is “yes”. If the patient indicates that he was not approached regarding inhaled medications or that he is not using inhaled medications, then the answer is “no”	Yes No
5.Has the patient been informed about the proposed medical treatment?		Ask the patient whether he was informed of the importance of maintaining the proposed medical treatment	If the patient received information about the need for continued medication use, then the answer is “yes”. If the patient did not receive information about the need for continued medication use, then the answer is “no”	Yes No
6.Was information provided regarding the treatment period for corticosteroid and antibiotic medications?		Ask the patient or the treating physician whether the patient was informed about the duration of corticosteroid or antibiotic medication use, if prescribed	If instructions were written on the discharge prescription or the patient was clearly told that correct use of antibiotic and corticosteroid medications was important, then the answer is “yes”. If the aforementioned information was not relayed, then the answer is “no”	Yes No
7.Was a follow-up scheduled with the attending physician or a pulmonologist?		Ask the patient whether he has a follow-up appointment scheduled with the attending physician or a pulmonologist after discharge	If a definite follow-up visit with the attending physician or a pulmonologist is scheduled, then the answer is “yes”. If no follow-up appointment is scheduled, then the answer is “no”	Yes No

COPD: chronic obstructive pulmonary disease; SpO_2_: oxygen saturation.

Fonte: Traduzido de Morais LA, Conde MB, Rabahi MF. Avaliação padronizada de pacientes com DPOC no momento da alta hospitalar. Rev Educ Saude. 2016;4(2):88-94; Quadro 3: Formulário padronizado para verificação dos itens propostos pela GOLD em pacientes com DPOC, no momento da alta hospitalar.

Pacientes que não completaram os procedimentos do estudo foram excluídos. Após entrevista de todos os indivíduos, os médicos responsáveis foram entrevistados em relação ao uso das diretrizes GOLD de alta hospitalar.

Os dados foram analisados utilizando o (SPSS), versão 23. Adotou-se nível de significância de 5% (p<0,05) na análise. O teste Shapiro-Wilk foi utilizado para avaliar a distribuição (normalidade) de variáveis numéricas. Os dados sociodemográficos e as respostas no documento foram caracterizados por frequências absolutas e relativas. Utilizou-se o teste de χ^2^ou teste exato de Fisher para avaliar as variáveis qualitativas como apropriados. As médias para variáveis quantitativas foram comparadas utilizando o teste *t* Student. O teste de confiabilidade foi realizado por meio da coleta de dados de documento utilizando o coeficiente kappa.

## RESULTADOS

Foram incluídos 63 pacientes no estudo. Destes, nove não concordaram em assinar o Termo de Consentimento. A amostra final incluiu 54 indivíduos (22 de hospitais com Programa de Residência Médica em Pneumologia e 32 de hospital sem o programa). As características sociodemográficas da amostra são mostradas na tabela 2 . Entre os 12 clínicos entrevistados sobre o uso das diretrizes GOLD de alta hospitalar, todos responderam “não”.

**Tabela 2:** Características sociodemográficas dos participantes do estudo

Característica	Total n=54	RMP n=22	Sem RMP n=32	Valor de p
Idade, anos	66,6±11,4	63,5±12,0	68,7±8,6	0,09
Sexo masculino	28 (51,9)	14 (61,9)	11 (34,4)	0,06
Educação				0,1
Sem escolaridade	8 (24,2)	6 (33,3)	2 (15,3)	
Fundamental	25 (75,8)	12 (66,7)	13 (86,7)	
Internação anterior em UTI	8 (14,8)	5 (19,0)	3 (9,4)	0,08
Readmissão	14 (42,4)	8 (44,4)	6 (40,0)	0,8

Resultados expressos como média±desvio padrão, ou n (%). RMP: programa residência médica em pneumologia; UTI: unidade de terapia intensiva.

Como demonstrado na tabela 3 , 75% (116/154) das respostas sobre as sete questões foram adequadas entre os pacientes que receberam alta hospitalar em unidade com Programa de Residência Médica em Pneumologia, e 47% (106/224) foram adequadas entre os pacientes que receberam alta de hospital sem o programa. Observaram-se diferenças significantes entre grupos para os itens 1, 4 e 7.

**Tabela 3:** Informação de tratamento e informação disponível aos pacientes com doença pulmonar obstrutiva crônica na alta hospitalar

Item avaliado	RMP Sim n=22 n (%)	Sem RMP Sim n=32 n(%)	Valor de p
1.Prescrição para uso de medicamentos para DPOC	22 (100)	21 (66)	0,002*
2.Gasometria arterial e avaliação da SpO_2_	22 (100)	30 (94)	0,2
3.Plano de conduta para comorbidades	6 (27)	12 (37)	0,4
4.Avaliação de técnica para uso de inaladores	20 (90)	18 (56)	0,001*
5.Informação em relação à manutenção da terapia	19 (86)	29 (90)	0,6
6.Introdução em relação a duração do corticoide ou terapia antibiótica	7(32)	15 (47)	0,2
7.Plano de seguimento por especialista	20 (90)	10 (32)	0,001*

* significativo.

RMP: programa residência médica em pneumologia; DPOC: doença pulmonar obstrutiva crônica; SpO_2_: saturação de oxigênio.

## DISCUSSÃO

Os achados deste estudo mostram que as respostas relacionadas à disponibilização de informação sobre tratamento e doença aos pacientes com DPOC na alta hospitalar, de acordo com a diretriz GOLD de alta hospitalar, foram geralmente mais correta entre aqueles de hospitais Programa Residência Médica em Pneumologia (75%) do que entre aqueles de hospital sem o programa (47%). Os menores níveis disponíveis para hospitais com Programa Residência Médica em Pneumologia, de acordo com pacientes e quadros, foram observados nos itens 3 (plano de conduta para comorbidades) e 6 (instruções em relação à duração de corticoide ou terapia com antibiótico), e menores níveis de informação disponibilizados por hospitais sem Programa Residência Médica em Pneumologia foram observados para os itens 3 (plano de conduta para comorbidades), 4 (avaliação da técnica para uso de inaladores), 6 (instruções em relação à duração de corticoide ou terapia antibiótica) e 7 (plano de seguimento com especialista). Essas recomendações foram levantadas pela GOLD em 2014 sendo mantidas e atualizadas no documento na versão 2019.

Os médicos entrevistados foram residentes que atenderam hospitais na clínica médica. Sugere-se fortemente adesão a protocolos com objetivo mais direto e assistência ao paciente padronizado para todas as categorias médicas.

A DPOC está associada a comorbidades como doença pulmonares e metabólicas que influenciam significativamente no prognóstico.^9^Spielmanns et al.,^10^mostraram que as comorbidades podem influenciar nos riscos de internação em UTI e ventilação mecânica. Um total de 73% dos pacientes de hospitais com Programa de Residência Médica em Pneumologia e de 63% daqueles de hospitais sem Programa de Residência Médica em Pneumologia não possuía planos de conduta. A falta de plano de conduta apropriada para comorbidades pode ter impacto negativo no tratamento, já que os pacientes hospitalizados com DPCO, em geral, apresentam comorbidades que levam a piora dos resultados a pacientes sem DPOC.^11^Um estudo mostrou que a readmissão pode ser evitada se as comorbidades forem melhores avaliadas durante a transição da alta hospitalar.^12^

O tratamento da DPOC depende do uso de inaladores multidose.^13^Em nosso estudo, 44% dos pacientes de hospitais Programa Residência Médica em Pneumologia e 10% dos pacientes de hospitais com Programa Residência Médica em Pneumologia não estavam familiarizados com uso correto de inaladores. De acordo com estudo conduzido no Texas, o uso prévio de inaladores dosimetrados reduziu a taxa de readmissão hospitalar entre 9% e 23% após 30 e 60 dias, respectivamente, quando os pacientes com DPOC foram instruídos em relação ao uso correto de inaladores no momento da alta hospitalar.^13^

No total, 68% dos pacientes com alta dos hospitais com Programa Residência Médica em Pneumologia e 53% dos pacientes dos hospitais sem o programa receberam informação inadequada sobre terapia com antibióticos e corticoides. As evidências indicam que os pacientes são geralmente incapazes de lembrar o diagnóstico ou o plano de tratamento na alta hospitalar.^14^A falta de informação, consequentemente, leva a altas taxas de readmissão hospitalar e a mais problemas na transição de paciente internados para assistência ambulatorial.^15^

Além disso, 10% dos pacientes de hospitais com Programa Residência Médica em Pneumologia e 68% de hospitais sem o programa tiveram seguimento ambulatorial agendado com pneumologista. A falta de acompanhamento é uma das variáveis associadas com readmissão hospitalar.^16^Parikh et al., demostraram o valor de disponibilizar informação para um grupo de pacientes.^17^Eles encontraram que 59,1% dos pacientes retornaram à clínica ambulatorial para visita com pneumologista, após serem instruídos na alta hospitalar para o fazerem.

As altas proporções de pacientes (100% e 90%, respectivamente) de hospitais com e sem o Programa Residência Médica em Pneumologia foram avaliadas para determinar a necessidade de oxigenoterapia por meio da verificação de gasometria arterial, resultados consistentes com os de Hernandez et al.^18^A oxigenoterapia de longo prazo tem sido efetiva para reduzir complicações em paciente com falha respiratória por hipoxemia, apesar de não terem sido identificadas diferenças na frequência de internação ou duração da permanência hospitalar antes, durante, e após a prescrição de oxigenoterapia.^19 , 20^

Este estudo tem diversas limitações principalmente relacionadas ao tamanho reduzido da amostra. O seguimento relativamente curto também constitui limitação que deve ser considerada no desenho de estudos futuros. Além disso, nossos resultados parecem ser aplicáveis somente a pacientes com características similares àqueles de nossa amostra.

## CONCLUSÃO

A falta de disponibilização de informação relacionada aos sete itens abordados pelas diretrizes *Global Initiative for Chronic Obstructive Lung Disease* aos pacientes com doença pulmonar obstrutiva crônica na alta hospitalar sugere que essas diretrizes devem ser utilizadas como rotina por clínicos em hospital com e sem Programa Residência Médica em Pneumologia. O verdadeiro impacto da adoção desta política deve ser avaliado por meio de abordagem intervencionista.
